# Simultaneous Mental Fatigue and Mental Workload Assessment With Wearable High-Density Diffuse Optical Tomography

**DOI:** 10.1109/TNSRE.2025.3551676

**Published:** 2025-03-14

**Authors:** Jianan Chen, Huixin Yang, Yunjia Xia, Tingchen Gong, Alexander Thomas, Jia Liu, Wei Chen, Tom Carlson, Hubin Zhao

**Affiliations:** HUB of Intelligent Neuro-Engineering (HUBIN), CREATe, Division of Surgery and Interventional ScienceUCL HA7 4LP London U.K.; School of Biomedical EngineeringThe University of Sydney 4334 Sydney NSW 2050 Australia

**Keywords:** Brain-computer interface, functional near-infrared spectroscopy, high-density diffuse optical tomography, mental fatigue, mental workload, support vector machines, random forest

## Abstract

Accurately assessing mental states—such as mental workload and fatigue— is crucial for ensuring the reliability and effectiveness of brain-computer interface (BCI)-based applications. Relying on signals from a limited brain region with low spatial resolution may fail to capture the full scope of relevant information. To address this, high-density diffuse optical tomography (HD-DOT), an emerging form of functional near-infrared spectroscopy (fNIRS) was employed in this study, which provides higher spatial resolution for hemodynamic measurements and enables the reconstruction of 3D brain images. An experiment protocol was designed to investigate both mental workload and fatigue, two critical components of cognitive state that often fluctuate concurrently in real-world scenarios. Machine learning methods were applied for subject-specific classification, achieving 95.14% mean accuracy for fatigue/non-fatigue and 97.93% for four n-back tasks using Random Forest, outperforming Support Vector Machines. These results highlight the transformative potential of HD-DOT in advancing multifaceted cognitive state assessment, paving the way for more precise, adaptable, and powerful BCI applications.

## I. Introduction

As a direct communication channel between brain activity and external devices, brain-computer interfaces (BCI), especially active/synchronous BCIs, have been increasingly used by motor-impaired patients for both control applications and rehabilitation [1]. While repeated training sessions are necessary and may reduce mental demand due to increased familiarity and task proficiency, maintaining consistent BCI performance remains challenging. Variations in performance are often attributed to psychological factors such as attention fluctuations and fatigue, especially during extended training sessions [2]. Consequently, understanding the user’s mental state is crucial in BCI applications, enabling better adaptation of control strategies or rehabilitation content to enhance their effectiveness [3]. For instance, detecting cognitive overload and fatigue in users of BCI-controlled smart wheelchair can improve operational reliability by adjusting control mechanisms to prevent errors [4]. Passive/asynchronous BCI systems offer a promising solution, enabling unobtrusive monitoring and interpretation of mental states through neural signals without requiring active input or intentional commands [5].

Mental Workload (MW) represents the proportion of an individual’s cognitive resources required to meet task demands [6]; perceived workload increases as task demands approach the cognitive capacity [5]. Factors influencing MW include task properties (e.g., difficulty), environmental conditions (e.g., noise), and individual characteristics (e.g., fatigue, cognitive capacity, training level, and emotional state) [5]. Most studies on MW in passive BCI contexts focus exclusively on workload without considering Mental Fatigue [7], [8]. However, in highly fatigued conditions, even simple tasks can become challenging, as MF impairs attention and increases the likelihood of behavioral lapses [9]. This reduction in cognitive capacity and sustained attention may decrease the operational efficiency of BCI-based systems, especially in assistive or rehabilitation devices [10], [11]. Thus, a comprehensive understanding of users/patients’ cognitive states, considering both task difficulty and fatigue levels, is essential for accurately assessing task performance and mitigating error risks in practical BCI applications. In this study, a comprehensive experiment protocol was designed to assess both MW and MF, utilizing varying levels of the n-back task [12] to induce fatigue.

Advancements in neuroimaging device and signal processing techniques have significantly improved the capability to monitor mental states including fatigue, emotion, and cognitive workload—driving their widespread application in human-passive BCI and robot interaction [13]. Two commonly used wearable functional imaging techniques in BCI include electroencephalography (EEG) and functional near-infrared spectroscopy (fNIRS). EEG captures electrical signals reflecting the activity of neuronal populations over a short duration through electrodes positioned on the scalp [1]. fNIRS, an optical imaging technique, measures hemodynamic changes in the brain by detecting the distinct absorption of near-infrared light by oxygenated (HbO) and deoxygenated hemoglobin (HbR) [14]. fNIRS tracks regional cerebral oxygenation changes, making it a valuable tool for studying mental states [15], with source localization of specific cortical regions being crucial in MW studies [16].

However, both EEG and traditional fNIRS systems (with around or less than 100 channels [17]) have inherent limitations in their source localization capabilities. EEG records electrical field changes on the scalp, but signal propagation through the scalp, skull, and brain tissues causes significant diffusion and attenuation, reducing spatial resolution. While traditional fNIRS provides millimeter-level spatial resolution [18], it is highly sensitive to hemodynamic responses in the shallow (extracerebral) tissue layer [19]. Short-channel regression methods using varying source-detector (S-D) distances have been proposed to reduce false positives from extracerebral signals [20]. However, relying on only two S-D separations limits these methods to two depth layers, hindering accurate 3D mapping of blood oxygenation changes.

As an extension of fNIRS, Diffuse Optical Tomography (DOT) employs varying S-D separations for overlapping spatial sampling, capturing depth-resolved, 3D images of cerebral hemodynamics and enhancing understanding of brain activity [21], [22]. A DOT array can be considered high-density DOT (HD-DOT) if it includes channels with at least three S-D separations, and the sensitivity distributions of these multi-distance channels overlap spatially to ensure continuous sampling of the underlying tissue [23]. HD-DOT enables functional neuroimaging of the cortex with a resolution comparable to functional Magnetic Resonance Imaging (fMRI), yet without requiring the immobilization necessary for fMRI [24], [25]. It has already demonstrated utility in cognitive and clinical research [23], establishing it as the optimal tool for this study.

Current fNIRS studies on MW achieve relatively high binary classification accuracy but often lack precise localization of brain activation, and few studies have successfully delivered robust multi-class accuracy [16]. For BCI applications, it is essential to maintain high performance across all users and scenarios. In this study, data collection was conducted using a wearable HD-DOT device [26] which covers the prefrontal cortex, offering the advantage of wearability and ease of setup. The study employed machine learning (ML) methods, including Support Vector Machines (SVM) [27] and Random Forest [28], to classify varying levels of MF and MW. These algorithms, well-suited for processing complex, high-dimensional data like HD-DOT signals, demonstrated robust performance and interpretability, outperforming most of existing studies.

Main contributions of this work include:

• Experimental Design: Successfully developed an experimental framework that simultaneously introduces varying levels of cognitive demand and induces fatigue, enabling a comprehensive investigation of MW and MF dynamics.
• High Classification Accuracy in n-Back Tasks: Demonstrated the ability of HD-DOT data to differentiate between distinct n-back task levels, achieving high four-class classification accuracy.
• Fatigue State Analysis and 3D Imaging: Achieved high binary classification accuracy for fatigue states before and after n-back tasks, while leveraging 3D imaging data to provide novel visualizations of mental states, enhancing understanding of the cognitive processes underlying fatigue during tasks.

**Fig. 1.**
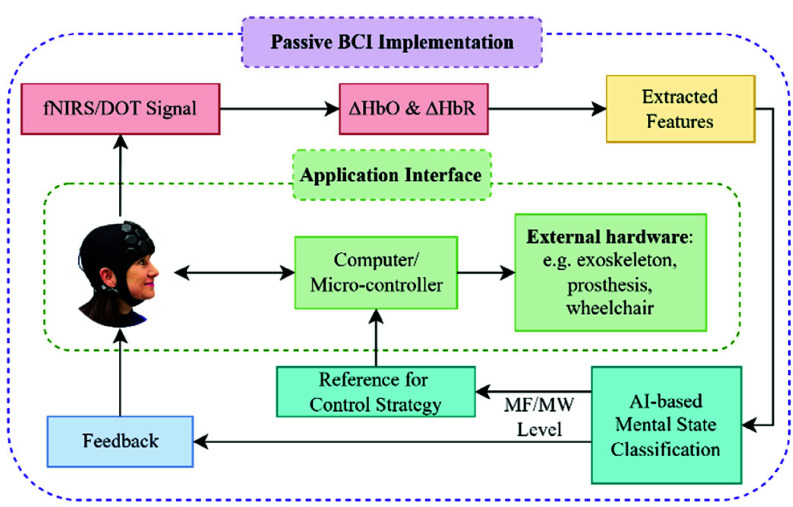
Scheme of application for AI-based mental state monitoring with HD-DOT.

This study underscores the potential of HD-DOT, combined with artificial intelligence (AI), as a practical and scalable solution for real-world cognitive state monitoring in BCI applications and beyond (Figure 1).

## II. Methods

### A. Participants Recruitment

Participants are required to be between 18 and 65 years old, possess normal or corrected vision and hearing, and have no history of neurological disease, disorder, injury, or cognitive impairment. We recruited 24 healthy participants (16 males, 8 females) with ethics approved by the institutional ethics committee (UCL REC ref: 6860.017; 6860.018). Their ages ranged from 20 to 30 years. All participants were right-handed.

### B. Experimental Paradigm and Procedure

**Fig. 2.**
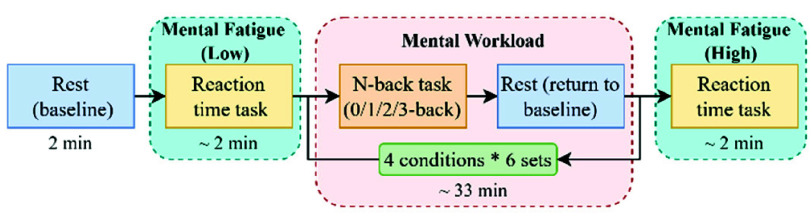
Experiment protocol. (Each MW task session includes the instruction, task, and rest block.)

**Fig. 3.**
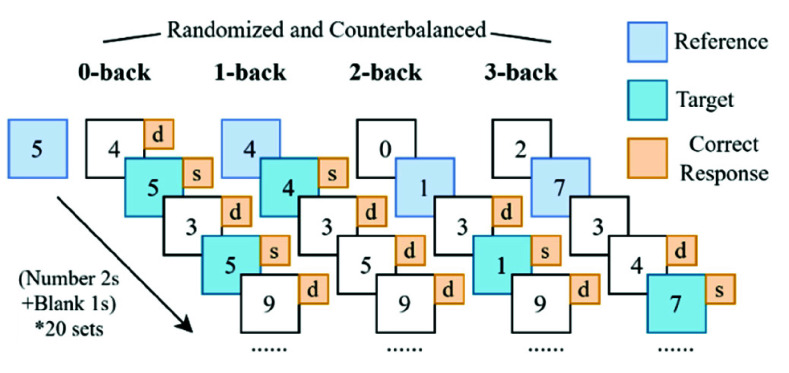
Explanation of n-back tasks for n $\in $ {0,1,2,3}. (Participants identified target numbers using key presses: ‘s’ for ‘same’ and ‘d’ for ‘different.’ 0-back: Participants respond to a specific target stimulus every time it appears, which is five in our experiment. 1-back: Participants respond when the current number matches the previous one; 2-back: Participants respond when the current one matches the number two steps back; 3-back: Participants respond when the current one matches the number three steps back.)

All the experiments were designed and implemented using PsychoPy (Open Science Tools Ltd., University of Nottingham) [29]. The experiment protocol consists of two main parts: Reaction Time (RT) task and n-back task [30], as shown in Figure 2. Participants first completed a 2-minute resting period to establish a baseline state. Subsequently, they performed RT task, which involved pressing the ‘space’ key promptly upon the appearance of a circle stimulus. The reaction time was recorded for further analysis. To induce MF, the n-back task was administered (Figure 2). Participants performed four specific tasks: 0-back, 1-back, 2-back, and 3-back, each having six sets and requiring comparison of the current number with previously presented numbers (Figure 3).

In each n-back task, stimuli were presented sequentially, and participants responded according to the rules outlined in Figure 3. The task difficulty increased with higher n-back levels, demanding more memory and attention. To minimize potential bias between English-native and non-native speakers, numbers were used instead of letters in the N-back task. Finally, participants completed another RT task.

### C. Data Collection and Device Description

The measurements for MW and MF can be divided into subjective and objective measures [31]. We included all these measures: subjective scores, task performance and HD-DOT data during the data acquisition.

HD-DOT data was collected using the wearable HD-DOT device LUMO (Gowerlabs Ltd, London, UK) [26] at wavelengths of 735 nm and 850 nm. The prefrontal cortex is the area of interest in most cognitive studies [32]. The 12-tile prefrontal configuration used in this study offers 1728 dual-wavelength channels (with usually $300\sim 500$ effective channels), with S-D separation varies from 10 mm (intra-tile) to 50 mm (cross-tile) [33]. Data collection was conducted using the Lumox software (Gowerlabs Ltd, London, UK [32]), and the entire experiment lasted approximately 40 minutes.

### D. Data Preprocessing

The recorded data includes subjective scores obtained from questionnaires, behavioral data collected through PsychoPy, and neurological measurements obtained via LUMO.

#### 1) HD-DOT Data Preprocessing:

**Fig. 4.**
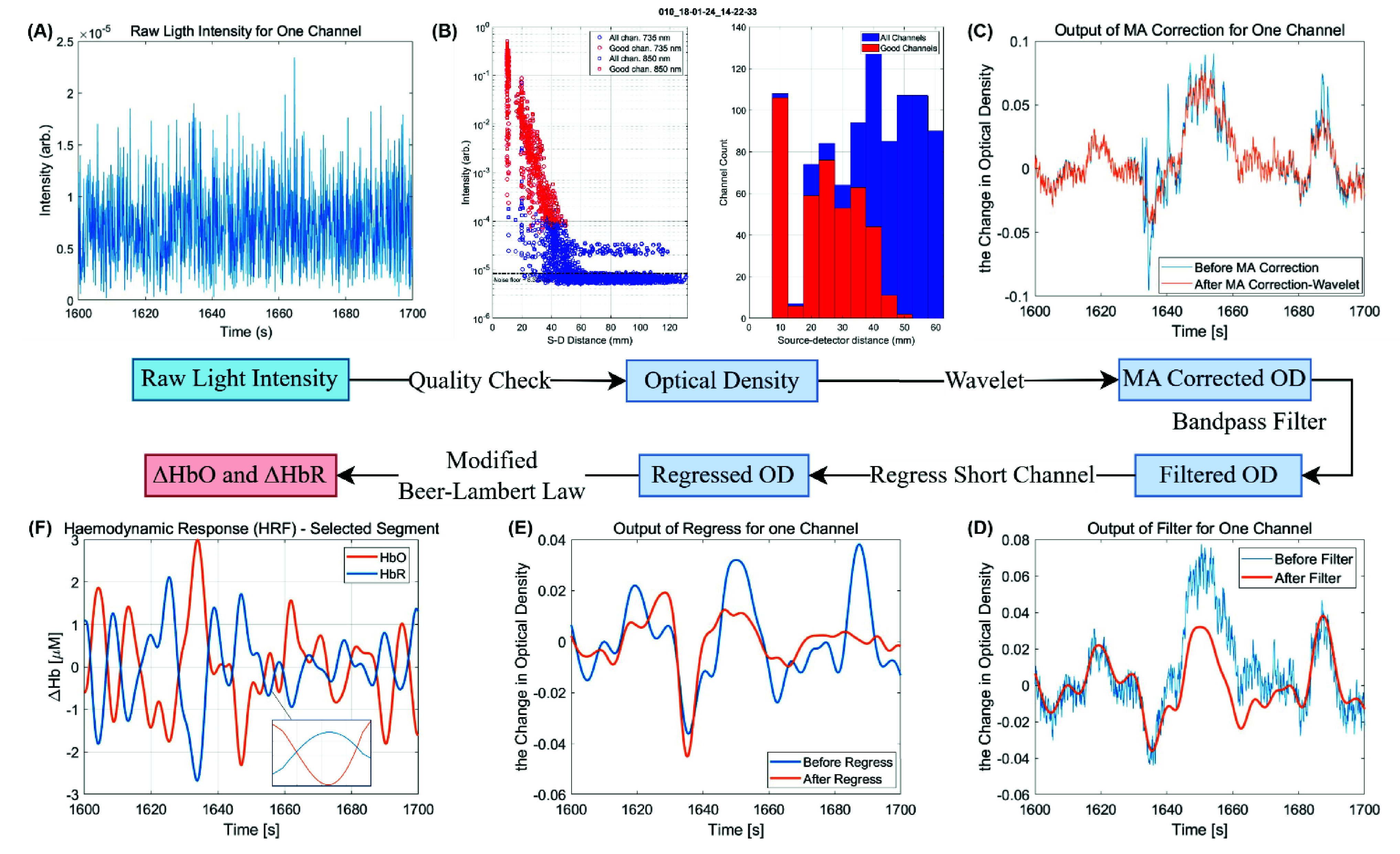
Flowchart of HD-DOT data preprocessing. The comparisons of thesignal before and after each processing step are plotted to help understand the role of each step. The six subfigures illustrate key stages of data processing, using selected data from a high-quality channel between 1600 and 1700 seconds from participant 010 as an example. A) Raw Light Intensity: The recorded raw light intensity for a single channel is displayed. This intensity represents the detected light after passing through biological tissue. B) Quality Check: This panel presents a quality assessment of all measurement channels. The left plot shows the distribution of raw intensity values as a function of distance, with “good” channels marked in red and “noisy” channels in blue. The right histogram categorizes the number of channels according to source-detector distances, differentiating between retained (good) and discarded (bad) channels. C) Motion Artifact Correction (Wavelet Method): The plot compares OD changes before (blue) and after (orange) motion artifact correction, showing reduced abrupt signal fluctuations. D) E) Short Channel Regression & Bandpass Filtering: The OD signal is further refined by removing systemic physiological noise using short-channel regression, followed by bandpass filtering to eliminate slow drifts and high-frequency noise. The filtered OD signal (orange) demonstrates improved smoothness and signal clarity compared to the unfiltered signal (blue). F) Final Hemodynamic Response ( $\Delta $HbO and $\Delta $HbR): The processed OD signal is converted into hemoglobin concentration changes using the modified Beer-Lambert law. A detailed processing pipeline can be found in the supplementary material (Figure 1).

The preprocessing of fNIRS data was performed using DOTHUB-Toolbox [34] in MATLAB 2022b. The acquired raw light intensity data was first preprocessed to ensure quality before being converted into optical density (OD), as shown in Figure 4. fNIRS signals are often impaired by various noise including instrumental, experimental, and physiological disruptions [14], [35]. Data containing motion artifacts were identified using standard deviation (STD) and amplitude (AMP) thresholds [36], then removed using a wavelet-based method [37]. A zero-phase Butterworth bandpass filter (0.025-0.15 Hz) was then applied using the hmrBandpassFilt function from the HOMER2 software package [38] to eliminate most physiological noise, such as heartbeat, respiration, and blood pressure fluctuations [35]. This was followed by short-channel regression to effectively eliminate superficial blood oxygenation changes from the scalp, isolating the relevant cerebral blood oxygenation changes. Finally, the OD was converted into relative HbO and HbR concentration levels (unit: $\mu $M) using the modified Beer-Lambert law [39].

#### 2) Behavioral and Subjective Data:

a) Behavioral data: PsychoPy software recorded timing, trial information, responses, accuracy, and experimental conditions. Our analysis focuses specifically on reaction time, defined as the interval between stimulus appearance and when participant response by pressing the key, as well as accuracy of different N-back tasks.
b) Subjective response: Participants rated their MF with modified Karolinska Sleepiness Scale (KSS) [40] on a scale from 0 (no fatigue) to 5 (maximum fatigue) under two conditions: initially, during the first RT task (prior to the n-back task), and subsequently, during the second RT task (following the n-back task). The detailed description of the modified KSS can be found in the supplementary materials (Table. II).

## III. Data Analysis

Statistical analysis was first conducted to validate the effectiveness of the experimental design and to obtain a general understanding of the HD-DOT data, which also facilitated feature selection. Subsequently, ML methods were employed to classify MW and MF states based on change in concentration of HbO and HbR signals.

### A. Validation of Fatigue and Workload Induction

To determine the effectiveness of the fatigue induction, we analyzed both objective measures (performance) and subjective measures (questionnaire ratings) of MF and different levels of MW.

A paired one-tailed t-test was conducted to determine whether there is a significant difference in self-report score before and after N-back. A t-value of −9.02 and p-value close to 0 indicate a statistically significant difference, which means the experiment protocol successfully induced a certain degree of tiredness (Figure 6A). Only two participants reported they feel the same in terms of alertness and clarity.

**Fig. 5.**
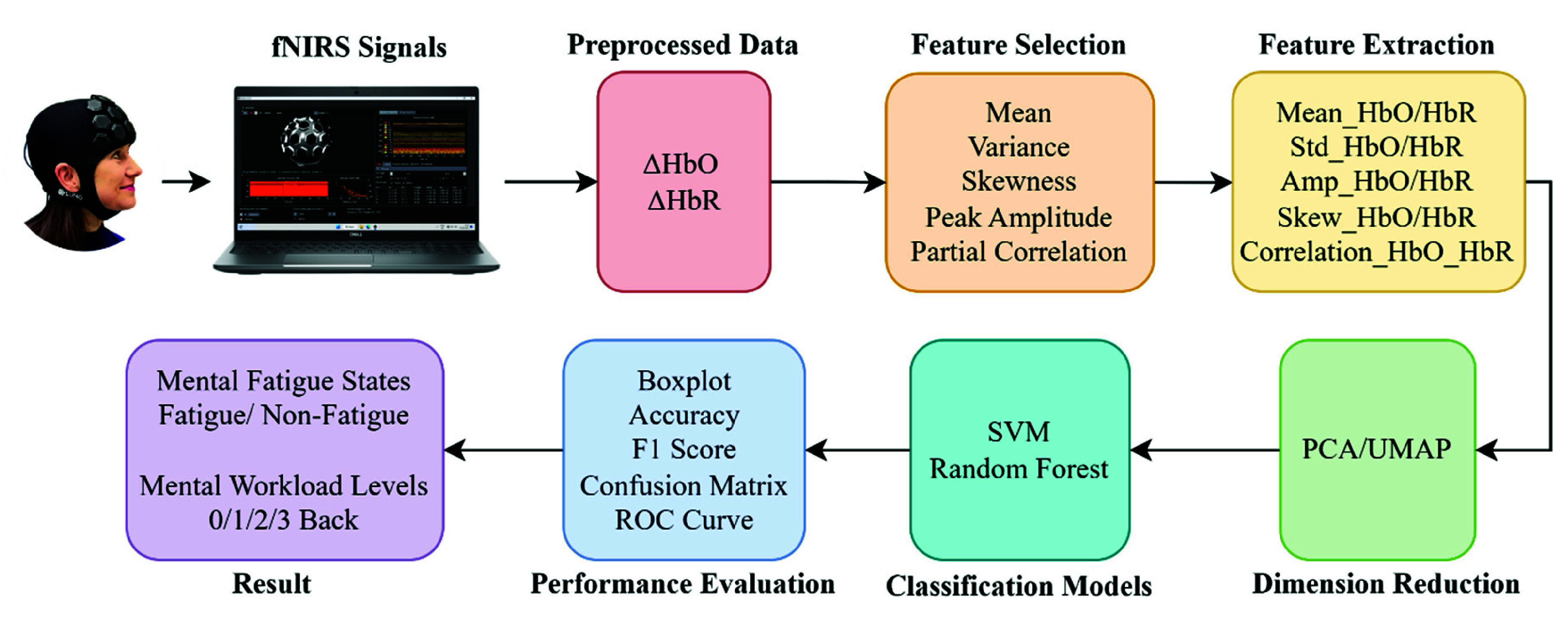
Workflow for MW and MF detection with ML.

**Fig. 6.**
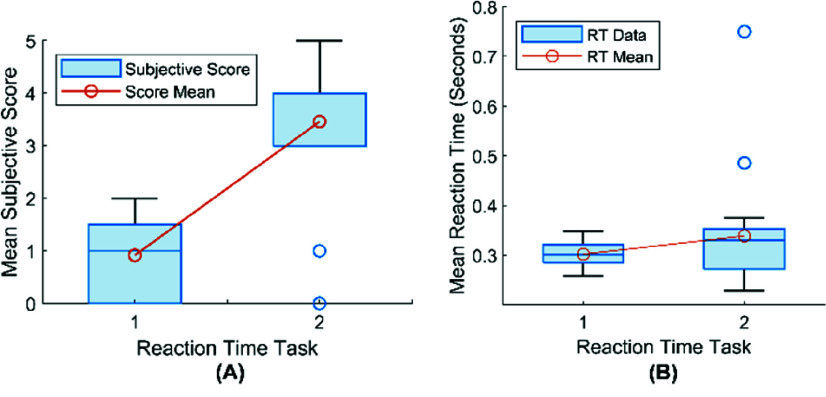
(A) Box plot for subjective score. (Each subject was asked to rate their Subjective Score before and after the n-back tasks, with scores ranging from 0 to 5 to indicate the level of MF.) (B) Box plot for mean reaction times (in seconds) of two RT tasks. (1^st^ RT has a slightly lower mean reaction time compared to 2^nd^, as indicated by the orange markers representing the mean reaction time for each task.)

For the first and second RT task in Figure 6B, mean reaction times are also displayed as box plots with individual outliers marked above the main distribution. The Shapiro-Wilk test indicates that the differences between the two groups do not follow a normal distribution (p-value: $4.38\times 10 ^{-7}$). Therefore, the Wilcoxon signed-rank test was used to compare the two groups and resulted in a p-value less than 0.05 (p-value: 0.034). Moreover, the 2^nd^ RT shows a slightly wider interquartile range, suggesting more variability in response times compared to the 1^st^ RT. This increase in mean and variability in 2^nd^ RT indicates a potential influence of fatigue cumulated during the N-back tasks, which could reduce the performance of participants in identical RT tasks.

**Fig. 7.**
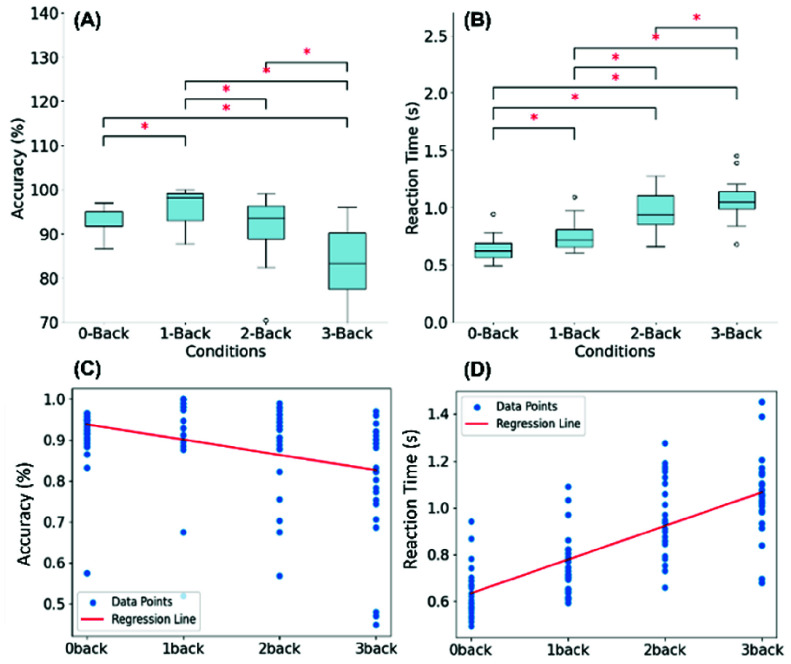
(A) Box plot of accuracy (%) and (B) Box plot of reaction time (s) across four different N-back task levels, with statistical significance markers (*). (C) Regression Analysis Results with N-back V.S. Reaction Time. (D) Regression Analysis Results with N-back V.S. Performance Accuracy.

We used t-test with Bonferroni correction for each pair of n-back. In Figure 7A, accuracy of responses is highest for the 1-back task (with a mean of 96.3%) and decreases as the N-back level increases, with the lowest accuracy observed in the 3-back condition (mean 83.2%). Statistical significance markers (*) above the bars indicate significant differences in accuracy between task levels, suggesting a progressive decrease in accuracy as cognitive load increases. Figure 7B illustrates reaction time increases with higher N-back levels, indicating that participants took longer to respond as task difficulty increased. The significance markers (*) highlight statistically significant differences in reaction time between conditions, reinforcing that reaction times are affected by the increasing memory demands of higher N-back levels.

From the statistic tests and regression analysis (Figure 7), we can see an inverse relationship between task difficulty and accuracy, along with a positive correlation between task difficulty and reaction time. These findings highlight the impact of cognitive load on performance in the N-back task and validate the effectiveness of the mental state induction protocol.

### B. Analysis of HD-DOT Data

Linear Mixed Model (LMM) is an advanced regression model designed for repeated measurements, incorporating both fixed and random effects to handle correlated and dependent data [18]. We use LMM to identify significant fixed effects prior to feature selection and classification with ML, while also evaluating the significance of random effects to account for inter-subject variability and ensure the robustness of our model. The results table can be found in the supplementary material file (Table III–VI).

From the LMM analysis of mean HbO and HbR using 0-back data as the baseline, the fixed effects yielded estimated coefficients for each predictor variable. The intercepts for HbR ( $0.001~\mu $M) and HbO ( $0.004~\mu $M) represent baseline values when all predictors are at their reference levels. Neither intercept was statistically significant, suggesting no meaningful deviation from zero. Task condition, reflecting task difficulty, emerged as a significant predictor of both HbR and HbO levels. Specifically, the 1-back condition demonstrated a significant decrease in HbR ( $- 0.003~\mu $M) and HbO ( $- 0.003~\mu $M) with p-values less than 0.001. Similarly, the 2-back condition showed a significant decrease in HbR ( $- 0.003~\mu $M) and HbO ( $- 0.004~\mu $M), also with p-values less than 0.001. In contrast, the 3-back condition displayed a modest increase in both HbR ( $0.002~\mu $M) and HbO ( $0.002~\mu $M), with significance levels of p <0.05 for HbR and p = 0.017 for HbO. The slight increase in hemodynamic responses at 3-back may reflect a non-linear interaction between task load, cognitive resource allocation, and individual capacity. Gender (coded as Female) was not a significant predictor. Finally, the group variance, representing random effects, was close to zero for both HbR and HbO. This result suggests minimal variability attributable to random effects, implying that the model’s explained variance is primarily captured by the fixed effects (n-back levels and gender).

Similar results can be seen from For LMM analysis of mean HbO and HbR using the rest data as the baseline. All conditions (0-back, 1-back, 2-back, 3-back) show highly significant effects (p <0.001) compared to the reference level, “rest”. Confidence interval of the intercept ( $- 0.026~\mu $M) includes 0, suggesting the baseline is not significantly different from zero. In contrast, using the rest period as the baseline reveals a stronger task effect compared to the 0-back task, with larger coefficients indicating greater task-related activation relative to rest.

The LMM model offers initial insights into HD-DOT signals but lacks participant-specific details and spatial information. Therefore, we next introduce how ML methods are employed to achieve precise classification.

### C. Feature Extraction and Classification

#### 1) Extraction of Features Related to Fatigue and Workload:

As shown in Figure 5, various features including mean, variance, skewness, peak amplitude, and correlation—were calculated. Subsequently, these features were concatenated and normalized using L2 normalization. Principal Component Analysis (PCA) and Uniform Manifold Approximation and Projection (UMAP) [41] were then applied [42], reducing the dimensionality of the data to four components (in the case of SVM) and 20 components (in the case of Random Forest) for further analysis. Both methods were examined, and PCA was finally used. HD-DOT signals typically contain noise and redundancy, and PCA excels at extracting dominant features, offering good performance while maintaining a lower computational cost.

#### 2) Model Building and Validation:

The model training process for MW and MF was conducted separately for each participant, employing a five-fold outer cross-validation strategy to assess generalization performance. The algorithms were implemented using scikit-learn package [43] with Python 3.11. In each fold, parameter tuning was performed using a two-step approach. Initially, RandomizedSearchCV was used to explore a broad range of hyperparameters for both the SVM and RandomForest models. For SVM, the hyperparameters included the regularization parameter C, the kernel coefficient $\gamma $, and the choice of kernel function, which could be ‘linear’, ‘polynomial (poly)’, ‘radial basis function (rbf)’, or ‘sigmoid’. For RandomForest, the focus was on the number of estimators, tree depth, minimum samples for splitting, minimum samples for leaf, and the criterion for splitting. A total of 50 iterations were run in the randomized search across five inner cross-validation folds to identify the best preliminary hyperparameters. Once the optimal hyperparameters were found using RandomizedSearchCV, a more refined search was conducted using GridSearchCV, focusing on the parameters identified during the initial search. The grid search involved a fixed parameter grid based on the best results from the randomized search and further optimized the model’s hyperparameters.

The final model, after grid search refinement, was used to predict the test set in each of the five folds. The results were stored and a comprehensive classification report, including accuracy, F1 scores for each iteration and the best parameters can be found in the supplementary materials.

## IV. Classification Results

### A. Results From HD-DOT Data

#### 1) Mental Fatigue:

**Fig. 8.**
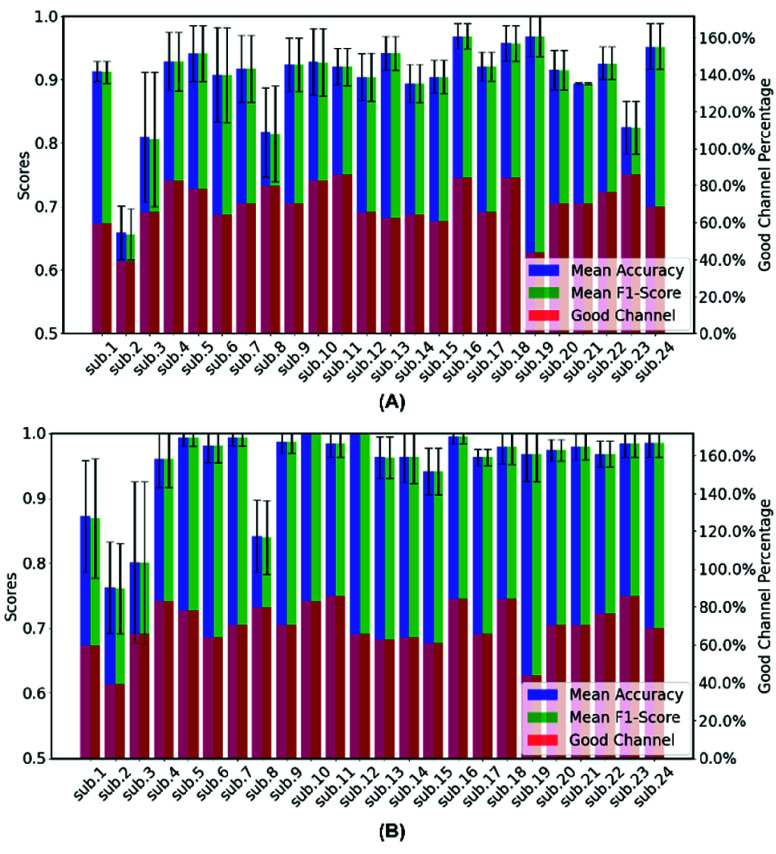
(A) Mean accuracy and (B) mean F1-score from 5-fold validation for MF with SVM and Randon forest. (The red bar indicates percentage of good quality channels in the S-D range of 27.5mm~32.5mm.)

HD-DOT data from the two different reaction time periods was classified into two categories: low/no fatigue and high fatigue. Figure 8 presents the performance of SVM across 24 participants, with mean accuracy and mean F1-score used as evaluation metrics. Both metrics demonstrate good performance, with mean accuracy values ranging from approximately 66% to 97% across participants. The relatively lower classification performance for participant 2 may be attributed to data quality issues, as analysis revealed fewer satisfactory channels for this participant. This is likely due to the participant’s exceptionally dense, dark hair, which may have partially obstructed some optodes, imitating near-infrared light penetration and compromising signal quality. The results of Random Forest are better overall, with most of the mean accuracy exceeding 90%. The Mean F1-score also closely follows the trend of mean accuracy, which suggests that the model’s classification precision and recall are well balanced across participants.

#### 2) Mental Workload:

SVM and Random Forest were used to classify four different workload levels.

**Fig. 9.**
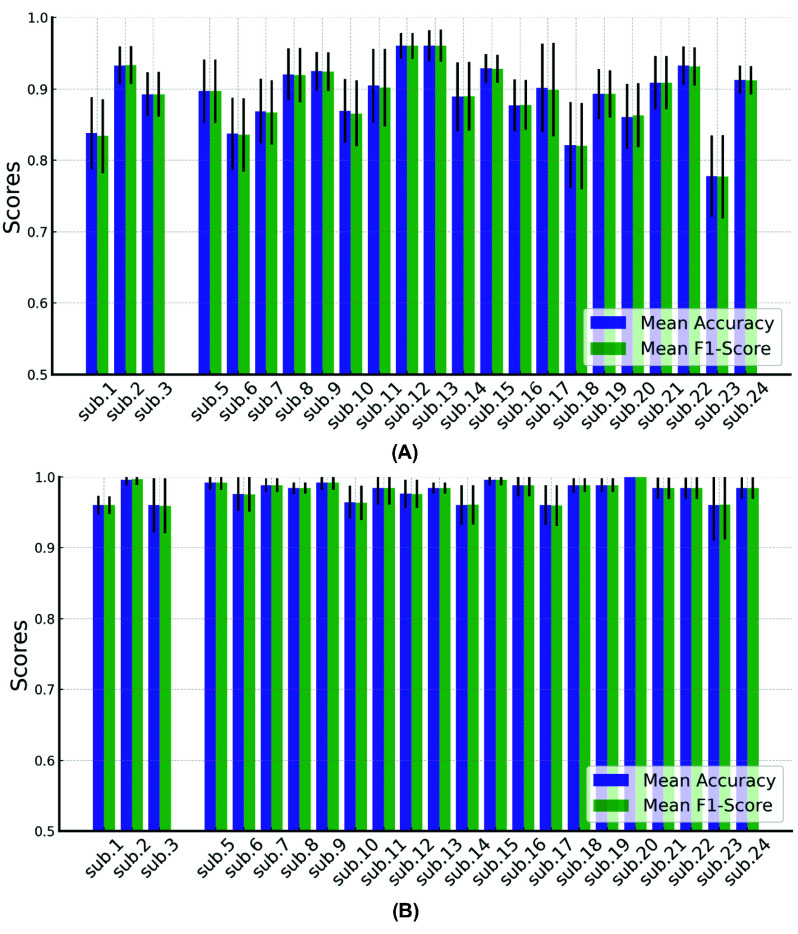
(A) Mean accuracy and (B) mean F1-score from 5-fold validation for N-back with SVM and Randon forest.

For the SVM model, the scores for both accuracy and F1 consistently remain above 0.7. This indicates that the model performs well overall, demonstrating strong generalization across the datasets. Additionally, certain participants such as 18 and 20 appear to have lower mean values for both metrics when compared to the rest. The Random Forest classifier has shown a significantly better performance, with the majority of accuracy and F1 scores exceeding 0.9 (Figure 9). Data from participants 4 were removed as the n-back data was contaminated with artifacts. The STDs of Random Forest method are generally smaller, suggesting that the model demonstrates stable performance across different data subsets.

### B. Performance Evaluation of Classification Models

**Fig. 10.**
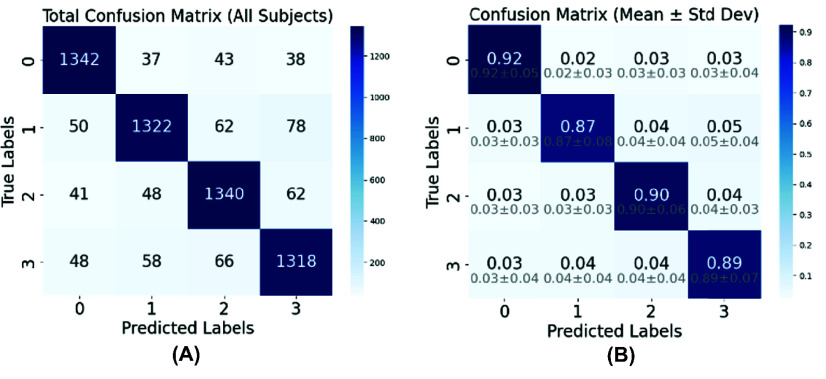
Mean accuracy and mean F1-score for N-back.

Confusion matrices were generated for the SVM model to facilitate understanding and interpretation by identifying misclassified instances and highlighting categories that may be challenging to classify, as shown in Figure 10. The standard confusion matrix shows the raw counts of predictions for each label. Misclassifications are relatively low, but there are noticeable misclassifications between neighboring classes (e.g., 3-back misclassified as 2-back), suggesting possible overlap in feature representations. These can be attributed to the inherent similarity in cognitive demands between the 2-back and 3-back tasks. These tasks both involve high working memory loads, leading to overlapping neural activation patterns that challenge the classifier’s ability to distinguish between them. The normalized confusion matrix with mean ± STD values reveals that 2-back exhibits the highest level of misclassification. It highlights the relative consistency of classification performance across cross-validation folds (low STD in diagonal values).

### C. Results for Groupwise Imaging

**Fig. 11.**
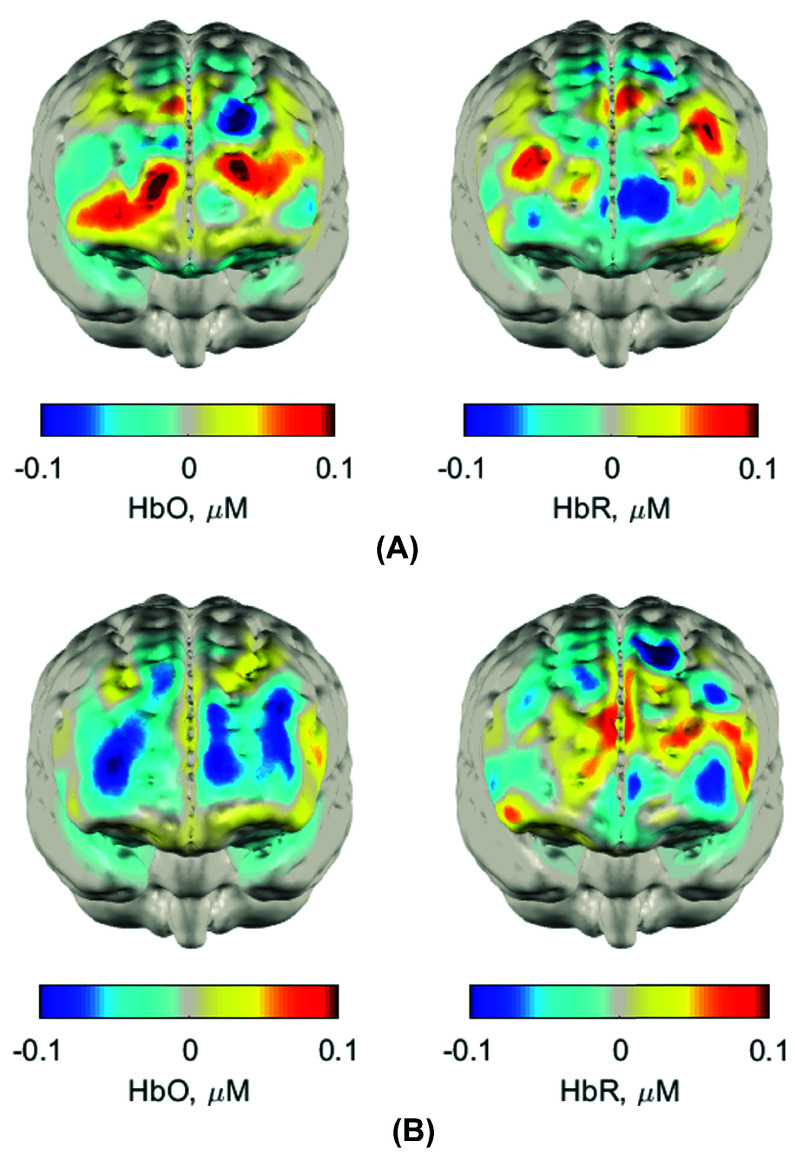
Groupwise 3D-DOT imaging results under (A) low/no and (B) high fatigue conditions.

Figure 11 illustrates group-level changes in cerebral oxygenation during the 1^st^ and 2^nd^ RT task. In the low/no fatigue state (Figure 11A), increased HbO, particularly in regions associated with task-related activation, suggests enhanced neuronal activity. The areas of increased HbO correspond with decreased HbR, reflecting the expected dynamic coupling of oxygen delivery and consumption during cognitive engagement. This pattern highlights a balance between neural activation and metabolic demand, characteristic of a well-functioning, low-fatigue state [44].

Conversely, in the high fatigue state (Figure 11B), HbO levels are reduced across most regions, indicating diminished cerebral blood flow. Concurrently, HbR concentrations are elevated, particularly in previously activated areas. This shift reflects the physiological impact of fatigue, where the brain struggles to sustain activation, leading to decreased engagement and metabolic inefficiency [45].

## V. Discussion and Future Work

### A. Discussion of Main Findings

The primary objectives of this study can be divided into three components: (i) to develop an experimental design capable of simultaneously introducing varying levels of cognitive demand and inducing fatigue throughout the process; (ii) to evaluate the ability of HD-DOT data to differentiate between distinct n-back task levels; and (iii) to classify fatigue states before and after the n-back tasks, which are utilized as MF inducers, and to analyze 3D imaging data to investigate fatigue states during the performance of a RT task.

i) Significant differences were observed in task accuracy and response times across n-back levels. Questionnaire results indicated that most participants experienced fatigue and perceived distinct differences in task difficulty, underscoring the effectiveness of our experimental design.
ii) HD-DOT data during n-back tasks were analyzed using both statistical regression modeling and ML methods. LMM analysis revealed that task difficulty (n-back conditions) significantly influenced HbO and HbR levels. SVM, commonly used for fNIRS-BCI signal classification due to its ability to handle high-dimensional data and robust theoretical foundation [46], was initially applied to classify the data. While overall performance was satisfactory, with most accuracy exceeding 80% and a mean of 89.2% for the four-class task, confusion matrices revealed challenges in distinguishing n-back tasks with similar difficulty levels, likely due to overlapping brain activation patterns. Among the tested kernel functions, the RBF kernel achieved the best performance, reflecting the nonlinear and high-dimensional nature of HD-DOT data. Additionally, higher STDs in accuracy and F1 scores during 5-fold validation suggest variability across trials, possibly caused by the complexity of HD-DOT data and the large number of channels, which may influence hyperplane placement and result in unstable classification boundaries.
  To address this, Random Forest was explored as an alternative, leveraging its ensemble approach to mitigate the impact of noisy or anomalous data while providing robustness and computational efficiency. Classification using Random Forest yielded higher mean accuracy than SVM (all mean accuracy exceeding 96%), outperforming many of EEG-BCI studies (92.9% for the 2-class and 80.3% for the 4-class n-back tasks in [47]) and fNIRS-BCI studies (84.3% in [19], 93.33% in [8]), demonstrating its suitability for MW classification with HD-DOT data. Comparison of relevant publication can be found in Table IX in the supplementary materials.
iii) A similar ML analysis was conducted on RT task data, yielding a mean accuracy of 90.1% with SVM and 95.1% with Randon Forest across all participants. The classification performance was lower compared to the MW task, likely due to the variability in participants’ fatigue levels upon arrival. Questionnaire scores and task performance indicated that most participants felt fatigued and performed worse during the in the 2^nd^ RT task. However, two participants reported no noticeable change in fatigue levels, suggesting that the 35-minute n-back task duration may have been insufficient to induce consistent MF across all participants. To better understand task performance under varying fatigue levels, 3D images of HbO and HbR were plotted on a group level.

In Figure 11(B), larger blue areas in the HbO plots indicate reduced brain activity in the high-fatigue state. This observation aligns with previous studies reporting a decline in activity within task-specific brain regions, such as the middle frontal gyrus (MFG), as fatigue progresses [45]. Additionally, prior research has demonstrated that activation in specific brain regions, including the anterior cingulate cortex, insula, and MFG, plays a critical role in regulating cognitive effort during task performance. The observed decrease in HbO concentration in our results may reflect a diminished regulatory function under fatigue, impairing the brain’s ability to efficiently allocate cognitive resources.

Notably, a complementary relationship between HbO and HbR dynamics was observed, especially in Figure 11(A). Under normal conditions during cognitive tasks, such as reaction time tests, the anterior region of the prefrontal cortex (near the forehead) exhibited significant activation. This was characterized by an increase in HbO levels, accompanied by a corresponding decrease in HbR levels within the same region, consistent with the expected hemodynamic response function [48].

### B. Directions for Future Research & Practical Implications

We acknowledge that our sample is limited to healthy right-handed individuals aged 20–30, which may affect the generalizability of our findings. More diverse demographics, encompassing different age groups and potentially patient populations will be included in future studies.

Regarding the experimental protocol, within-subject experiments could be a promising direction for improvement, where the same participants undergo different fatigue induction protocols (e.g., prolonged cognitive tasks vs. real-life fatigue conditions). This approach would allow us to assess the robustness of models across diverse fatigue states and determine its ability to generalize across controlled laboratory-induced fatigue and naturally occurring fatigue in real-world scenarios. Furthermore, by analyzing individual variability in fatigue responses, we aim to refine our model to better capture subject-specific fatigue dynamics, ultimately enhancing its applicability in real-time BCI systems.

Research has shown that converting fNIRS time-series data into images enhances classification accuracy in cognitive tasks [49]. Building on this foundation, future studies will focus on three key directions to maximize the potential of HD-DOT data.

First, to better utilize spatial information, we aim to generate and analyze subject-specific 3D brain hemodynamics reconstructed from HD-DOT data. This approach offers deeper insights into the physiological mechanisms behind MF and MW, improves ML interpretability through intuitive visualizations, and enhances cognitive state classification. As deep learning methods continue to advance in the field of NIR/DOT [50], [51], we will further investigate their effectiveness in processing the 3D imaging generated. Second, integrating these high-resolution reconstructions into real-time systems will enable dynamic monitoring and feedback for BCI applications. Finally, another important focus is to establish reliable reference values for cognitive load related to fatigue. Real-time cognitive load feedback enables clinicians to adjust task difficulty or duration, ensuring optimal engagement and preventing overexertion. It also enhances progress tracking, allowing for tailored rehabilitation strategies.

This study on mental states using HD-DOT holds potential not only for advancing BCI applications [52] but also for broader applications. For mental health monitoring, HD-DOT identifies neuromarkers and tracks fatigue linked to conditions such as depression [53] and anxiety [54]. In high-risk settings, it facilitates real-time monitoring of mental states, mitigating risks in tasks like driving [9] and aviation [55], thereby ensuring safety and performance.

## VI. Conclusion

This study advances our understanding of mental states in a passive BCI setting by leveraging the unique capabilities of HD-DOT technology. By combining subjective scores, task performance metrics (accuracy and reaction time), and HD-DOT data, we achieved a comprehensive assessment of MW and MF. We employed both channel-wise analysis and an imaging-based approach to assess hemodynamic responses. In the channel-wise analysis, we calculated $\Delta $HbO and $\Delta $HbR from individual source-detector separations using the modified Beer-Lambert law. The extracted features were then directly input into the machine learning model for classification. HD-DOT provides high channel density with depth sensitivity, enabling accurate classification of mental states (over 97% for MW and over 95% for MF). Moreover, we utilized TOAST++ for image reconstruction, generating spatial maps of $\Delta $HbO and $\Delta $HbR. The generation of 3D brain imaging offered insight into neural activation patterns, identifying specific brain regions involved and their dynamic changes under high fatigue states. These spatially resolved maps of neural activity underscore HD-DOT’s potential to enhance both the granularity and interpretability of cognitive state assessments.

In the future, we aim to explore the interplay between MF and other BCI tasks further and to develop a real-time monitoring system leveraging HD-DOT’s capabilities for dynamic feedback, paving the way for more adaptive and effective BCI applications.

## Supplementary Materials

Supplementary Materials
