# Supplementary material for: Simultaneous Mental Fatigue and Mental Workload Assessment With Wearable High-Density Diffuse Optical Tomography
Source: IEEE Trans Neural Syst Rehabil Eng. 2025 Mar 14;33:1242–51. doi: 10.1109/TNSRE.2025.3551676 (PMC12270415; doi:10.1109/TNSRE.2025.3551676)
Supplement: Supplementary Materials [file supp5-3551676.docx]

Simultaneous Mental Fatigue and Mental Workload Assessment with Wearable High-Density Diffuse Optical Tomography

Jianan Chen, *Student Member, IEEE,* Huixin Yang, Yunjia Xia, *Student Member, IEEE*, Tingchen Gong, Alexander Thomas, *Student Member, IEEE,* Jia Liu, *Member*, *IEEE*, Wei Chen, *Senior Member,* *IEEE,* Tom Carlson, *Member, IEEE* and Hubin Zhao, *Member, IEEE*

TABLE I

Summary of Measurements for Mental Workload and Fatigue

|  | **Subjective measures** | **Objective measures** | |
| --- | --- | --- | --- |
| **Methods** | Subjective scales/ Questionnaires | Behavioral performance | Physiological measurements |
| **Pros/Cons** | Easy to implement. | Objective and quantifiable. | Real-time, sensitive, minimal task interference |
|  | Prone to biases; not real time or objective. | Context-dependent; Lacks real-time insight | High-cost instruments required |
| **Mental fatigue** | e.g. The Chalder Fatigue Scale [1] | Task accuracy;  Reaction time. | Brain activity (EEG, fNIRS) and peripheral measures (heart rate, eye movement, respiration, EMG, skin conductance) |
| **Mental workload** | Subjective workload assessment technique [2] |  |  |


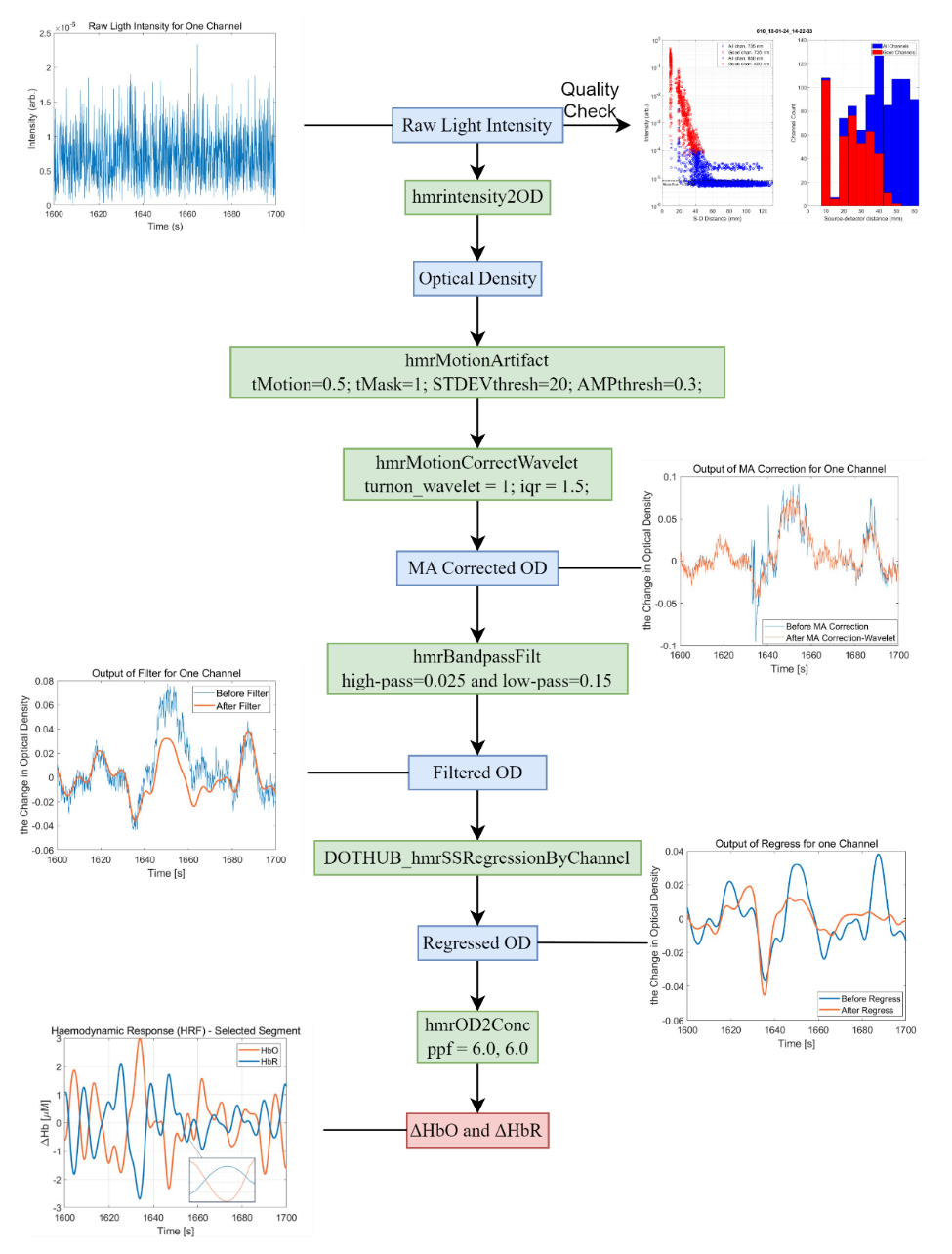


Figure 1. Flowchart of HD-DOT data preprocessing with detailed functions and parameters

Explanation for Fig.1:

This figure illustrates the step-by-step processing pipeline for HD-DOT data, transforming raw light intensity signals into hemoglobin concentration changes (ΔHbO and ΔHbR).

1. **Raw Light Intensity**: The recorded raw light intensity signal for a single channel is displayed. This intensity represents the detected light after it has passed through the biological tissue.
2. **Data Quality Check**: This function performs a source-detector distance check, motion artifact detection, and signal-to-noise ratio (SNR) pruning. It also visualizes all good channels in red against the source-detector distance.
3. **Conversion to Optical Density (OD)**: The raw intensity data is converted to optical density using the function hmrIntensity2OD. This step follows the formula *OD=log_10_(I_0_/I)*, where *I_0_* is the initial light intensity and *I* is the measured light intensity.
4. **Motion Artifact Detection**: Motion artifacts (MA) are identified using the function hmrMotionArtifact, which detects abrupt signal changes based on predefined thresholds (tMotion=0.5, tMask=1, STDEVthresh=20, AMPthresh=0.3).
5. **Motion Artifact Correction**: Identified motion artifacts are corrected using a wavelet-based approach (hmrMotionCorrectWavelet). A comparison of OD changes before and after motion artifact correction is shown.
6. **Bandpass Filtering**: A bandpass filter (hmrBandpassFilt) is applied to remove slow drifts and high-frequency noise, using cutoff frequencies of 0.025 Hz (high-pass) and 0.15 Hz (low-pass). The effect of filtering on OD changes is illustrated.
7. **Systemic Physiology Regression**: The function DOTHUB_hmrSSRegressionByChannel is used to remove global systemic influences, regressing out physiological noise from the OD signal. A comparison of OD changes before and after regression is shown.
8. **Conversion to Hemoglobin Concentrations**: The cleaned OD data is converted to hemoglobin concentration changes (ΔHbO and ΔHbR) using the function hmrOD2Conc, with a partial pathlength factor (PPF) of 6.0 for both wavelengths.
9. **Final Hemodynamic Response**: The processed hemodynamic response (HRF) for a selected segment is displayed, showing the final ΔHbO and ΔHbR signals used for further analysis.

TABLE II

A Description of the Modified Karolinska Sleepiness Scale (KSS)

| Alert | 1 |
| --- | --- |
| Rather alert | 2 |
| Some signs of fatigue | 3 |
| Tired but able to keep awake | 4 |
| Very tired, great effort to stay awake | 5 |

TABLE III

Results of Mixed Linear Model Regression with 0-back as Baseline (HbO)

| Model: | | MixedLM |  | Dependent Variable: | | HbO |  |
| --- | --- | --- | --- | --- | --- | --- | --- |
| No. Observations: | | 5636 |  | **Method:** |  | ML |  |
| No. Groups: | | 24 |  | **Scale:** |  | 0.0005 |  |
| Min. group size: | | 40 |  | **Log-Likelihood:** | | inf |  |
| Max. group size: | 336 | |  | **Converged:** | | Yes |  |
| Mean group size: | | 234.8 |  |  |  |  |  |
|  | | **Coefficient** | **Std. Error** | **Z Value** | **P>\|z\|** | **[0.025** | **0.975]** |
| Intercept | | 0.003648 | 19873.7 | 1.84E-07 | 1 | -38951.7 | 38951.74 |
| C(Condition, Treatment('rest'))[T.1back] | | -0.00283 | 0.000836 | -3.39205 | 0.000694 | -0.00447 | -0.0012 |
| C(Condition, Treatment('rest'))[T.2back] | | -0.00418 | 0.000836 | -5.00304 | 5.64E-07 | -0.00582 | -0.00254 |
| C(Condition, Treatment('rest'))[T.3back] | | 0.001987 | 0.000836 | 2.378314 | 0.017392 | 0.00035 | 0.003625 |
| Gender[T.Female] | | -0.02489 | 13997.07 | -1.78E-06 | 0.999999 | -27433.8 | 27433.72 |
| Group Var | | 0 |  |  |  |  |  |

TABLE IV

Results of Mixed Linear Model Regression with 0-back as Baseline (HbR)

| Model: | MixedLM |  | Dependent Variable: | | HbR |  |
| --- | --- | --- | --- | --- | --- | --- |
| No. Observations: | 5636 |  | Method: |  | ML |  |
| No. Groups: | 24 |  | Scale: |  | 0.0004 |  |
| Min. group size: | 40 |  | Log-Likelihood: | | inf |  |
| Max. group size: | 336 |  | Converged: | | Yes |  |
| Mean group size: | 234.8 |  |  |  |  |  |
|  | **Coefficient** | **Std. Error** | **Z Value** | **P>\|z\|** | **[0.025** | **0.975]** |
| Intercept | 0.000504 | 17719.72 | 2.84E-08 | 1 | -34730 | 34730 |
| C(Condition, Treatment('rest'))[T.1back] | -0.00263 | 0.000745 | -3.5365 | 0.000405 | -0.0041 | -0.00117 |
| C(Condition, Treatment('rest'))[T.2back] | -0.00342 | 0.000745 | -4.58723 | 4.49E-06 | -0.00488 | -0.00196 |
| C(Condition, Treatment('rest'))[T.3back] | 0.001507 | 0.000745 | 2.022235 | 0.043152 | 4.64E-05 | 0.002967 |
| Gender[T.Female] | 0.007941 | 12480.01 | 6.36E-07 | 0.999999 | -24460.4 | 24460.38 |
| Group Var | 0 |  |  |  |  |  |

TABLE V

Results of Mixed Linear Model Regression with Rest Period as Baseline (HbO)

| Model: | MixedLM |  | Dependent Variable: | | HbR |  |
| --- | --- | --- | --- | --- | --- | --- |
| No. Observations: | 8664 |  | Method: |  | ML |  |
| No. Groups: | 23 |  | Scale: |  | 0.0879 |  |
| Min. group size: | 361 |  | Log-Likelihood: | | inf |  |
| Max. group size: | 496 |  | Converged: | | Yes |  |
| Mean group size: | 376.7 |  |  |  |  |  |
|  | **Coefficient** | **Std. Error** | **Z Value** | **P>\|z\|** | **[0.025** | **0.975]** |
| Intercept | -0.026 |  |  |  |  |  |
| C(Condition, Treatment('rest'))[T.0back] | -1.634 | 0.010 | -170.604 | 0.000 | -1.653 | -1.616 |
| C(Condition, Treatment('rest'))[T.1back] | -1.636 | 0.010 | -170.808 | 0.000 | -1.655 | -1.617 |
| C(Condition, Treatment('rest'))[T.2back] | -1.638 | 0.010 | -170.980 | 0.000 | -1.657 | -1.619 |
| C(Condition, Treatment('rest'))[T.3back] | -1.632 | 0.010 | -170.318 | 0.000 | -1.650 | -1.613 |
| Gender[T.Female] | 0.134 | 114093.118 | 0.000 | 1.000 | -223618.268 | 223618.536 |
| Group Var | 0.000 |  |  |  |  |  |

TABLE VI

Results of Mixed Linear Model Regression with Rest Period as Baseline (HbR)

| Model: | MixedLM |  | Dependent Variable: | | HbR |  |
| --- | --- | --- | --- | --- | --- | --- |
| No. Observations: | 8664 |  | Method: |  | ML |  |
| No. Groups: | 23 |  | Scale: |  | 0.0562 |  |
| Min. group size: | 361 |  | Log-Likelihood: | | inf |  |
| Max. group size: | 496 |  | Converged: | | Yes |  |
| Mean group size: | 376.7 |  |  |  |  |  |
|  | **Coefficient** | **Std. Error** | **Z Value** | **P>\|z\|** | **[0.025** | **0.975]** |
| Intercept | 0.064 |  |  |  |  |  |
| C(Condition, Treatment('rest'))[T.0back] | -1.287 | 0.008 | -168.032 | 0.000 | -1.302 | -1.272 |
| C(Condition, Treatment('rest'))[T.1back] | -1.289 | 0.008 | -168.262 | 0.000 | -1.304 | -1.274 |
| C(Condition, Treatment('rest'))[T.2back] | -1.290 | 0.008 | -168.401 | 0.000 | -1.305 | -1.275 |
| C(Condition, Treatment('rest'))[T.3back] | -1.285 | 0.008 | -167.740 | 0.000 | -1.300 | -1.270 |
| Gender[T.Female] | 0.038 | 91226.053 | 0.000 | 1.000 | -178799.740 | 178799.816 |
| Group Var | 0.000 |  |  |  |  |  |

TABLE VII

Results of Mean Accuracy and Mean F1-score for Mental Fatigue and Mental Workload with SVM

| Subject | MF | | MW | | |
| --- | --- | --- | --- | --- | --- |
|  | Mean_Accuracy | Mean_f1 | | Mean_Accuracy | Mean_f1 |
| 1 | 0.913±0.016 | 0.911±0.017 | | 0.837±0.051 | 0.834±0.052 |
| 2 | 0.658±0.043 | 0.656±0.040 | | 0.933±0.026 | 0.933±0.026 |
| 3 | 0.810±0.102 | 0.806±0.106 | | 0.893±0.031 | 0.892±0.031 |
| 4 | 0.929±0.046 | 0.928±0.046 | | - | - |
| 5 | 0.941±0.044 | 0.941±0.044 | | 0.897±0.044 | 0.897±0.044 |
| 6 | 0.907±0.075 | 0.907±0.075 | | 0.837±0.051 | 0.835±0.052 |
| 7 | 0.917±0.052 | 0.917±0.053 | | 0.869±0.045 | 0.867±0.045 |
| 8 | 0.817±0.070 | 0.814±0.075 | | 0.920±0.036 | 0.919±0.038 |
| 9 | 0.923±0.042 | 0.923±0.042 | | 0.924±0.027 | 0.924±0.027 |
| 10 | 0.928±0.053 | 0.927±0.053 | | 0.869±0.045 | 0.865±0.046 |
| 11 | 0.920±0.029 | 0.920±0.029 | | 0.904±0.051 | 0.902±0.054 |
| 12 | 0.904±0.037 | 0.904±0.037 | | 0.960±0.018 | 0.960±0.018 |
| 13 | 0.941±0.027 | 0.941±0.027 | | 0.960±0.022 | 0.960±0.022 |
| 14 | 0.893±0.030 | 0.893±0.030 | | 0.889±0.048 | 0.890±0.048 |
| 15 | 0.905±0.026 | 0.904±0.026 | | 0.929±0.020 | 0.928±0.020 |
| 16 | 0.968±0.020 | 0.968±0.020 | | 0.877±0.036 | 0.878±0.035 |
| 17 | 0.920±0.023 | 0.920±0.023 | | 0.901±0.062 | 0.899±0.065 |
| 18 | 0.957±0.028 | 0.957±0.028 | | 0.821±0.060 | 0.820±0.061 |
| 19 | 0.968±0.032 | 0.968±0.032 | | 0.893±0.035 | 0.893±0.033 |
| 20 | 0.915±0.031 | 0.915±0.031 | | 0.861±0.046 | 0.863±0.045 |
| 21 | 0.894±0.001 | 0.893±0.001 | | 0.909±0.037 | 0.909±0.037 |
| 22 | 0.926±0.026 | 0.926±0.026 | | 0.932±0.027 | 0.932±0.027 |
| 23 | 0.825±0.041 | 0.824±0.042 | | 0.777±0.057 | 0.777±0.058 |
| 24 | 0.952±0.036 | 0.952±0.036 | | 0.913±0.020 | 0.912±0.020 |
| Average | 0.901±0.039 | 0.901±0.039 | | 0.892±0.039 | 0.891±0.039 |

TABLE VIII

Results of Mean Accuracy and Mean F1-score for Mental Fatigue and Mental Workload with Randon Forest

| Subject | MF | | MW | | |
| --- | --- | --- | --- | --- | --- |
|  | Mean_Accuracy | Mean_f1 | | Mean_Accuracy | Mean_f1 |
| 1 | 0.872±0.086 | 0.869±0.092 | | 0.960±0.013 | 0.960±0.012 |
| 2 | 0.762±0.070 | 0.761±0.069 | | 0.996±0.008 | 0.996±0.008 |
| 3 | 0.801±0.124 | 0.800±0.125 | | 0.960±0.038 | 0.959±0.039 |
| 4 | 0.960±0.044 | 0.960±0.044 | | - | - |
| 5 | 0.993±0.013 | 0.993±0.013 | | 0.992±0.010 | 0.992±0.010 |
| 6 | 0.980±0.026 | 0.980±0.026 | | 0.976±0.023 | 0.976±0.024 |
| 7 | 0.994±0.013 | 0.994±0.013 | | 0.988±0.010 | 0.988±0.010 |
| 8 | 0.842±0.056 | 0.839±0.056 | | 0.984±0.008 | 0.984±0.008 |
| 9 | 0.986±0.017 | 0.986±0.017 | | 0.992±0.010 | 0.992±0.010 |
| 10 | 1.000±0.000 | 1.000±0.000 | | 0.964±0.023 | 0.964±0.024 |
| 11 | 0.984±0.0.21 | 0.984±0.0.21 | | 0.984±0.023 | 0.984±0.023 |
| 12 | 1.000±0.000 | 1.000±0.000 | | 0.976±0.019 | 0.976±0.020 |
| 13 | 0.963±0.032 | 0.962±0.032 | | 0.984±0.008 | 0.984±0.008 |
| 14 | 0.963±0.040 | 0.963±0.040 | | 0.960±0.028 | 0.961±0.28 |
| 15 | 0.941±0.036 | 0.941±0.036 | | 0.996±0.008 | 0.996±0.008 |
| 16 | 0.995±0.011 | 0.995±0.011 | | 0.988±0.016 | 0.988±0.016 |
| 17 | 0.963±0.013 | 0.963±0.013 | | 0.960±0.028 | 0.960±0.029 |
| 18 | 0.979±0.026 | 0.979±0.026 | | 0.988±0.010 | 0.988±0.010 |
| 19 | 0.968±0.042 | 0.968±0.042 | | 0.988±0.010 | 0.988±0.010 |
| 20 | 0.973±0.017 | 0.973±0.017 | | 1.000±0.000 | 1.000±0.000 |
| 21 | 0.979±0.020 | 0.979±0.020 | | 0.984±0.015 | 0.984±0.015 |
| 22 | 0.968±0.020 | 0.968±0.020 | | 0.984±0.015 | 0.984±0.015 |
| 23 | 0.984±0.021 | 0.984±0.021 | | 0.960±0.051 | 0.960±0.049 |
| 24 | 0.984±0.021 | 0.984±0.021 | | 0.984±0.015 | 0.984±0.015 |
| Average | 0.951±0.032 | 0.951±0.032 | | 0.980±0.017 | 0.980±0.017 |

TABLE IX

Comparison of Mental Workload Related Publications and Proposed Method

| **Publication** | **Number of channels** | **Algorithm** | **Accuracy** |
| --- | --- | --- | --- |
| Lim et al., 2020 [3] | 52 channels with S-D distance of 30 mm | SVM | highest: 83.3%,74.3%, and 84.3% (for classification of low, medium, and high workloads) |
| Mughal et al., 2021 [4] | 36 channels | CNN-LSTM | 65.06% to 83.42% (0-, 2-, 3-back, or rest) |
| Park, 2023 [5] | 8 light sources and 2 light detectors | CNN | 0.83 to 0.96. (0-, 1-, 2-back) |
| Karmakar et al., 2023 [6] | 36 channels | CNN | Around 96% (2 classes) |
| Our proposed HD-DOT method | 1728 channels | SVM, Random Forest | 98.0% (0-, 1-, 2-, 3-back) |

References

[1] C. Jackson, “The Chalder Fatigue Scale (CFQ 11),” *Occup Med (Chic Ill)*, vol. 65, no. 1, pp. 86–86, Jan. 2015, doi: 10.1093/occmed/kqu168.

[2] G. B. Reid and T. E. Nygren, “The Subjective Workload Assessment Technique: A Scaling Procedure for Measuring Mental Workload,” 1988, pp. 185–218. doi: 10.1016/S0166-4115(08)62387-0.

[3] L. G. Lim *et al.*, “A unified analytical framework with multiple fNIRS features for mental workload assessment in the prefrontal cortex,” *IEEE Transactions on Neural Systems and Rehabilitation Engineering*, vol. 28, no. 11, pp. 2367–2376, Nov. 2020, doi: 10.1109/TNSRE.2020.3026991.

[4] N. E. Mughal, K. Khalil, and M. J. Khan, “FNIRS Based Multi-Class Mental Workload Classification Using Recurrence Plots and CNN-LSTM,” in *AIMS 2021 - International Conference on Artificial Intelligence and Mechatronics Systems*, Institute of Electrical and Electronics Engineers Inc., Apr. 2021. doi: 10.1109/AIMS52415.2021.9466084.

[5] J. H. Park, “Mental workload classification using convolutional neural networks based on fNIRS-derived prefrontal activity,” *BMC Neurol*, vol. 23, no. 1, Dec. 2023, doi: 10.1186/s12883-023-03504-z.

[6] S. Karmakar *et al.*, “Real time detection of cognitive load using fNIRS: A deep learning approach,” *Biomed Signal Process Control*, vol. 80, p. 104227, Feb. 2023, doi: 10.1016/j.bspc.2022.104227.
